# Improving child nutrition and development through community-based childcare centres in Malawi – The NEEP-IE study: study protocol for a randomised controlled trial

**DOI:** 10.1186/s13063-017-2003-7

**Published:** 2017-06-19

**Authors:** Aulo Gelli, Amy Margolies, Marco Santacroce, Katie Sproule, Sophie Theis, Natalie Roschnik, Aisha Twalibu, George Chidalengwa, Amrik Cooper, Tyler Moorhead, Melissa Gladstone, Patricia Kariger, Mangani Kutundu

**Affiliations:** 1 International Food Policy Research Institute (IFPRI), 2033 K Street NW, Washington, DC, 20006 USA; 2 Johns Hopkins University, Baltimore, MD USA; 3 Save the Children, USA/Malawi, Lilongwe, Malawi; 4 Ikapadata, RSA, Cape Town, South Africa; 5 University of Liverpool, Liverpool, UK; 6 University of California at Berkeley, Berkeley, CA USA; 7 Chancellor College, University of Malawi, Zomba, Malawi

**Keywords:** Preschool feeding, Impact evaluation, Nutrition, Agriculture, Child development

## Abstract

**Background:**

The Nutrition Embedded Evaluation Programme Impact Evaluation (NEEP-IE) study is a cluster randomised controlled trial designed to evaluate the impact of a childcare centre-based integrated nutritional and agricultural intervention on the diets, nutrition and development of young children in Malawi. The intervention includes activities to improve nutritious food production and training/behaviour-change communication to improve food intake, care and hygiene practices. This paper presents the rationale and study design for this randomised control trial.

**Methods:**

Sixty community-based childcare centres (CBCCs) in rural communities around Zomba district, Malawi, were randomised to either (1) a control group where children were attending CBCCs supported by Save the Children’s Early Childhood Health and Development (ECD) programme, or (2) an intervention group where nutritional and agricultural support activities were provided alongside the routine provision of the Save the Children’s ECD programme. Primary outcomes at child level include dietary intake (measured through 24-h recall), whilst secondary outcomes include child development (Malawi Development Assessment Tool (MDAT)) and nutritional status (anthropometric measurements). At household level, primary outcomes include smallholder farmer production output and crop-mix (recall of last production season). Intermediate outcomes along theorised agricultural and nutritional pathways were measured. During this trial, we will follow a mixed-methods approach and undertake child-, household-, CBCC- and market-level surveys and assessments as well as in-depth interviews and focus group discussions with project stakeholders.

**Discussion:**

Assessing the simultaneous impact of preschool meals on diets, nutrition, child development and agriculture is a complex undertaking. This study is the first to explicitly examine, from a food systems perspective, the impact of a preschool meals programme on dietary choices, alongside outcomes in the nutritional, child development and agricultural domains. The findings of this evaluation will provide evidence to support policymakers in the scale-up of national programmes.

**Trial registration:**

ISRCTN registry, ID: ISRCTN96497560. Registered on 21 September 2016.

**Electronic supplementary material:**

The online version of this article (doi:10.1186/s13063-017-2003-7) contains supplementary material, which is available to authorized users.

## Background

The 2013 *Lancet* series on Maternal and Child Nutrition estimated that the aggregate global burden of undernutrition causes over three million child deaths per year. Stunting prevalence in children under 5 years also affects at least 165 million children [1]. Recent reviews of the contribution of agriculture in improving nutrition [2, 3] conclude that although agricultural programmes have immense potential to improve nutrition, this potential is yet to be unleashed. Current evidence suggests that limitations in the design, targeting and implementation of agricultural interventions, as well as their lack of clarity in nutrition goals and the exact interventions which are being provided, are partly responsible for this weak evidence base [3].

Early Childhood Health and Development (ECD) programmes are designed to improve young children’s survival, growth and development. They are considered the most cost-effective form of human capital investment when compared to any subsequent schooling interventions [4]. ECD investments can increase the efficiency of ongoing public spending in education, in turn improving the overall allocation of public resources [5]. A recent review of ECD programmes, which included both efficacy trials and programme evaluations, has demonstrated how improving diets of pregnant women and young children can prevent stunting and result in enhanced cognitive and motor development of children [6, 7]. The most effective programmes provided activities targeted to younger and disadvantaged children that were integrated with health and nutritional services. Providing services to children directly and involving parents in practice and skill-building sessions were more effective strategies than providing information alone. Further evidence shows that the quality of the child’s learning environments, including in the home and in preschools, has a strong impact on child development. The potential for additive and synergistic effects of combining ECD, and nutrition, and agriculture and nutrition is clear [1]. Combining these three sectors may lead to substantial gains in cost, efficiency and effectiveness but these need to be rigorously evaluated.

The Nutrition Embedded Evaluation Programme Impact Evaluation (NEEP-IE) study is designed to evaluate the impact of an integrated package of nutritional and agricultural interventions (including improved nutritious food production and behaviour-change communication related to food intake, care and hygiene practices) on the diets, nutrition and development of young children and their households in rural areas of Malawi.

This paper presents the rationale and study design for this randomised trial.

### Country context

Malawi ranks 73rd out of 104 countries on the Global Hunger Index [8] and has one of the highest rates of chronic malnutrition in the world with 37% of children aged 6–59 months being moderately or severely stunted [9]. It is also one of the most committed countries to improving nutrition, ranking 3rd on the Hunger and Nutrition Commitment Index (HANCI). In March 2011, the Malawi Government joined the Scaling Up Nutrition (SUN) Movement promoting a multisectoral approach to tackling malnutrition. Malawi is one of the few countries that meet the African Union’s Maputo Declaration to spend over 10% of public expenditure on agriculture. An intersectoral coordinating body (National Nutrition Committee) was set up to support cross-sectoral integration and the Department of Nutrition has been placed in the Office of the President and Cabinet to highlight the government’s commitment to nutrition.

The national ECD programme, led by the Ministry of Gender, Children and Social Welfare (MoGCSW) targets all children aged 0–8 years. Preschools (known as community-based childcare centres (CBCCs)) and parenting groups are the two main components of the government ECD programme. CBCCs are community-initiated and community-owned childcare centres which aim to promote holistic child development by providing safe, stimulating environments, access to health and nutritional services, and training for parents and caregivers. Today, there are an estimated 9000 CBCCs across Malawi, serving 32%percent of all 3–5 year olds in the country. Parenting groups are typically linked to the CBCC and aim to reach parents of children aged 0–8 years, as well as parents to be (newly married and pregnant women).^1^

One of the main challenges facing CBCCs is lack of food (mid-morning porridge) [10]. The absence of food at the CBCCs is one of the main causes of child absenteeism and CBCC closure and the MoGCSW is looking for cost-effective ways to address the food gap in CBCCs. In the past 2 years, Save the Children, driven by demand from the CBCC communities it supports, began integrating nutritional, agricultural and livelihood components into its ECD programme. The goal is to help communities provide nutritious food to CBCCs all year round and improve food security and child nutrition at household level at the same time. The programme draws learnings from the USAID-funded Wellness and Agriculture for Life Advancement (WALA) project implemented across two districts for 5 years and the Conrad N Hilton Foundation-funded “Improving CBCC Meals” project implemented in over 200 CBCCs across four districts. Anecdotal evidence from the 68 communities currently benefiting from the integrated nutritional, agricultural/livelihood and ECD package suggests that CBCCs, CBCC gardens and parenting groups provide a very effective, and yet untested, platform for improving agricultural production, food security, child nutrition and caregiving practices at household level. As Save the Children scales up this integrated package to CBCCs currently supported in Zomba district, the impact evaluation will assess the feasibility, cost-effectiveness and impact on indicators relevant to each sector (nutrition, agriculture and ECD).

The Government of Malawi also recognises the need for multisectoral programming, particularly relating to nutrition [11] and is looking for evidence-based models to guide their national policies and strategies. The proposed intervention supports a number of Malawi’s national targets, most importantly those for nutrition, food security, education and child development and brings together three sectors at community level to implement a multisectoral, integrated, synergistic package adapted to the Malawian context.

### The standard ECD package

The standard package provided to Save the Children-supported communities includes the following activities. For the CBCC activities:

- CBCC caregivers receive a 2-week training, provided by government-approved trainers, using a standard government-approved training manual when they start. They receive 5-day refresher trainings annually and they are mentored by one visit/month by ECD facilitators
- CBCCs target all 3–5 year olds in the community. They are managed by the community and are open from 8 a.m. to 11 a.m., 5 days per week. Porridge (mid-morning meal) is provided at about 10 a.m. by parents/facilitators with food contributions from the community

For parenting education:

- CBCC caregivers and mentors received a 3-day training to organise parenting sessions using a standard government parenting education manual
- The parenting sessions are organised once or twice a month on average. Topics covered by parenting sessions include: child nutrition, stimulation, and parental role in school readiness, amongst others. Parenting sessions target all parents of children aged 0–8 years old with particular focus on children from 0–2 years
- Agricultural extension workers received a 5-day training to organise parenting sessions using a standard government parenting education manual
- Trainings were conducted between April and May 2016. The total number of people trained was 437 with 382 women and 55 men
- Other ECD interventions include children’s corners, mobile phones and interactive radio instructions (IRI)

### The integrated agricultural and nutritional intervention

The NEEP-IE integrated nutritional and agricultural intervention is aimed at improving the diets, feeding, health and hygiene knowledge and practices in households with infants and young children. This includes promoting optimal feeding and caring practices through parenting groups; engaging parents, adolescents and other adults in the community in the planning and preparation of meals for children within CBCCs; improving agricultural production of nutritious foods and food diversification by using CBCC gardens as a learning site for communities; forming Village Savings and Loans (VSL) groups to help communities save and access funds to purchase supplies for the CBCC garden and CBCC meals or to start new home-gardens; and by organising farmers into collectives to increase their purchasing and selling power.

More specifically, the agricultural training includes:

- Training on nutritious food production, including the following topics: production of foods to use in CBCCs, i.e. production of selected cereals and staples (orange maize, orange-fleshed sweet potato and cassava); selected legumes and nuts (soya beans, pigeon peas, cowpeas and groundnuts); and green leafy vegetables (*Amaranthus*). In all this the subtopics included: land preparation and planting, weeding and fertiliser application, pests and disease control, harvesting, storage and processing and utilisation in CBCCs. The use of manure as fertiliser was also promoted
- The CBCC garden was used as a demonstration site. At a demonstration plot, the community prepared the gardens with guidance from the Agriculture Extension District Officers (AEDOs). An AEDO would start a ridge with the correct spacing and the community would take over after that with the AEDOs making sure that the correct spacing is used and the correct type of ridge is made. This would continue until the rest of the garden has been prepared. The same process was adopted for all the gardens. Where the demonstration coincided with the first rains, the planting was also done, again with guidance from the AEDOs. AEDOs and community agents (CAs) monitor the gardens two to three times per month on average
- The first agricultural production training was facilitated by AEDOs and involved 900 participants in total. Each training session lasted 3 days and included 45 participants. The training took place in December 2015 and farmers (households) began planting soon after the training. The training was two-fold: theory and practice. A second agricultural production training was also facilitated by AEDOs to 120 participants. Each training session lasted 3 days and each session had 20 participants. These trainings took place in August 2016 and covered the following topics: land preparation and planting, weeding and fertiliser application, pests and disease control, harvesting, storage and processing and utilisation in CBCCs for carrots, spinach and tomatoes

In addition, participating households received a range of agricultural inputs as summarised in Table 1.

**Table 1:** Agricultural inputs provided to households as part of the agricultural and nutritional intervention

Seeds provided	Planting period	Harvest period	Consumption period
Orange maize	December	March-April	May onwards
Pigeon peas	December	April	April onwards
Cow peas	December	March	March onwards
Beans	December		
Groundnuts	December		
Soya	December	April	April onwards
OFSP	January	April-March	April onwards
Carrots	July	October	October onwards
*Amaranthus*	August	August/September	August onwards

*OFSP* orange-fleshed sweet potato

The nutritional training activities included:

- Training CBCC management committee members, caregivers, lead farmers and parents on the nutritional needs of children, healthy meals all year round, food selection, storage and preservation, food hygiene, safety and preparation, waste disposal, hand-washing, meal planning and monitoring of meal provision and recipes for CBCCs and households
- Each training session lasted 3 days and was provided by AEDOs and nutrition assistants The training combined both theory and practice, including cooking demonstrations. The participants were divided into groups of three to five people. Trainers would introduce selected recipes for nutritious meals, with instructions written on a card that was left with the group. The trainer would facilitate each step of the process. Thereafter, the groups would come together and display what they had cooked. At the display table, a member from each group was selected to explain how they made the meal, how many types of food groups the recipe contained, any alternatives and, lastly, answer any questions from the group. After the display, the participants tasted the food that they had made. The recipes included:
  1. Porridges: maize flour porridge with groundnut powder, maize flour porridge with dry fish powder, maize flour porridge with dry vegetables, mango porridge, porridge made from a mixture of maize, soya, millet, beans and groundnuts flour, rice porridge with carrot and oil, and rice porridge with groundnut flour
  2. Milk and soya milk production
  3. Legume products: pigeon peas sausage, cassava *kidos* (boiled cassava dipped in eggs and vegetables and fried), soya coffee, sweet potato doughnuts, peanut butter and soya snacks
  4. Vegetable products: pumpkin leaf meatballs (pumpkin leaves, salt and eggs as ingredients); orange-fleshed sweet potato (OFSP) juice, sweet potato leaf juice, dried vegetables (for preservation); pumpkin leaves and *Amaranthus* in groundnut powder and sweet potato leaf snack
  5. Fish products: dry fish with groundnut powder, dry fish with tomato and onion
  6. Fruit products: pawpaw, guava and lemon juice; pawpaw relish (unripe pawpaw cooked with groundnut powder, tomato and onion) and banana bread
- Trainings were conducted in February 2016 and CBCC caregivers/communities began putting into practice what they had learned from March 2016. CBCC caregivers and parents were then mentored and supervised by community-based organisations (CBOs) with four visits per month

### Study aim and objectives

The purpose of the NEEP-IE study is to provide evidence on the effectiveness and costs of delivering an integrated agricultural and nutritional intervention through CBCCs and parenting groups on the diets, nutrition and development of young children in rural areas of Malawi. The findings of the evaluation will be used to inform the Government of Malawi and development partners on the effectiveness and feasibility of scaling-up the intervention. The objectives of the evaluation include:

1. Evaluating the impact of integrating nutrition and agriculture with ECD on the diets, nutrition and development of children aged 36 to 72 months
2. Evaluating the impact of integrating nutrition and agriculture with ECD on CBCC meal provision, CBCC attendance and enrolment
3. Identifying the main factors that influence the impact of the intervention on child- and household-level outcomes
4. Evaluating the effectiveness of CBCCs and parenting groups as entry points to improve nutrition-related outcomes to other critical age groups, including younger siblings of preschoolers
5. Evaluating the cost, feasibility and sustainability of scaling-up the integrated nutritional and agricultural package through CBCCs and parenting groups

## Methods

### Programme theory

**Fig. 1:**
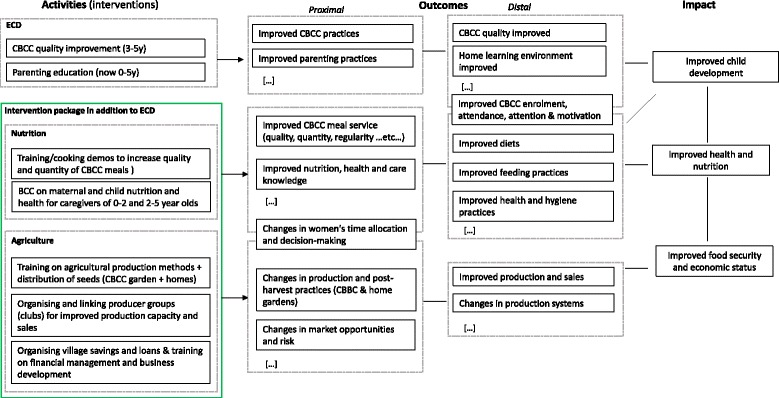
Overall programme theory for the agricultural and nutritional intervention

The overall programme theory for the package of nutritional and agricultural interventions is guided by the *Lancet s*eries framework on Maternal and Child Nutrition [1] and broadly summarised in Fig. 1. For a more detailed analysis of the complex pathways linking agriculture and nutrition, including the different processes, actors, effects and lags, see [2, 12–14]. The package of interventions affects health and nutrition directly by improving diets and feeding practices through the behaviour-change communication and nutritional education. This, in turn, has an indirect impact on preschooling, as improving health and nutrition has a positive impact on attention, cognition and learning. By increasing the regularity and quality of the CBCC meals the interventions will also directly influence children’s participation in the CBCC. The interventions can also affect agriculture by increasing production, sales and profits, and changing the crop production mix.

### Impact on children’s nutrition and health

The three determinants of undernutrition in children include food, health and care practices [1]. The main channels through which the intervention has an impact on nutrition and health is through improved diets via increased consumption of nutrient-rich foods, and through improved nutrition, health and hygiene practices.

The proposed intervention package can potentially have an impact on the nutrition and health of children enrolled in CBCCs and their younger siblings, as summarised in Fig. 3 in the Appendix. This involves a combination of direct transfers to the CBCC children (e.g. transfer of nutritious food through preschool meals) and indirect channels involving the behaviour-change campaigns promoting the consumption of nutritious foods and improved nutrition practices at household level. Improved diets, when accompanied by adequate feeding, health and hygiene practices can then contribute to improved health and nutrition. In particular:

- The nutritional impact is mediated by the extent of food substitution effects within the household, and the use of the nutritional intake by the child and their siblings
- Diversifying diets and increasing the intake of micronutrient-rich foods can have direct effects on attention and cognition
- Healthier and better-nourished children are then better positioned to learn new skills both at the CBCC and elsewhere
- All these effects are mediated to some degree by women’s roles in the household, time allocation and decision-making

Substitution, or intrahousehold reallocation, may occur when households readjust consumption patterns in response to the CBCC meals, or to a change in diet, by substituting foods normally consumed at home, or with other foods with similar properties. Substitution is a complex issue involving changing household dynamics where gender plays a fundamental role. Influencing possible substitution effects will be critical in determining the potential impacts on children’s nutrition and health.

Changes in individual-level dietary diversity have been found to be strongly associated with micronutrient adequacy of diets for women [15, 16] and micronutrient density adequacy of diets in children [15]. Addressing micronutrient deficiencies can improve a range of health, nutritional and developmental outcomes in infants and young children, particularly if implemented alongside behaviour change on health and nutritional practices [17]. Conceptually, this framework suggests that the emphasis of the interventions in the short term should focus on integrating nutritional education and messaging, alongside the CBCC meals, to deliver improved intake of micronutrient-rich foods, with the potential of leading to improved household diets.

Restoring micronutrients and enhancing energy intake can also have an impact on attention and motivation. Energy [18] and iron intake [19] can have an impact on hyperactivity, withdrawal, nervousness, hostile behaviour and happiness. The emotional status of children may also affect attention span and have other positive spill-overs. Caregivers and peers are also likely to be affected by the increase in attention and concentration.

### Impact on agriculture

From an agricultural perspective, the intervention focusses on increasing food production in the CBCC garden and home-gardens by increasing yields or efficiencies through input provisions or training on farming practices. The intervention can also influence the basket of products that are being produced, supporting the production of higher-value crops and/or more nutritious crops through the provision of seeds, educational campaigns, or the opening of new market channels. The selection of particular crops involves balancing the pros and cons of substitution between crops for production, sale and consumption and the long-term impacts for both incomes and nutrition (see [20] for more details).

### Main hypotheses and outcome indicators

The expected impact of the intervention discussed in the analysis of the programme theory is summarised below. The intervention is expected to have a positive impact on:

- Preschool enrolment and attendance
- Children’s diets and household diet diversity
- Infant and young children’s nutritional status and childcare practices
- Agricultural production

A limited impact is expected on:

- Micronutrient status, child development, physical growth
- Agricultural income

The main indicators for the evaluation are summarised in Table 2.

**Table 2:** Main outcome indicators of the intervention

Type	Domain	Indicators
Primary	Diets	Individual intake and diet diversity score (children 36–72 m)
Primary	Childcare practices	WHO IYCF practices
Primary	CBCC participation	CBCC enrolment and attendance (children 36–72 m)
Primary	Agriculture	Production output, crop-mix
Secondary	Health and nutritional status	Anthropometry (weight-for-age, height-for-age, weight-for- age z-scores and MUAC) (children 6–72 m)
Secondary	Child development	Malawi Development Assessment Tool z-scores (fine motor, gross motor, language and social domains) (children 36–72 m)
Secondary	Gender	Women’s asset ownership, time use and productivity
Process	Meal service	Quality of CBCC meals, portion sizes, frequency

*CBCC* community-based childcare centres, *IYCF* Infant and Young Child Feeding, *MUAC* mid-upper arm circumference, *WHO* World Health Organization

Note that in addition to outcome indicators we will also observe the programme impact on intermediate indicators, particularly for those outcomes that are more difficult to observe directly. In the agricultural domain, we will look at intermediate outcomes such as input use (labour, land, seeds and fertiliser), investments (farm capital, such as tools and machinery) and market access (marketed surplus, prices and markets). The quantity, quality and timely preparation and delivery of food in the CBCCs will also be explored.

### Design of the randomised evaluation

A cluster randomised trial (CRT) is being implemented in 60 rural communities with CBCCs supported by Save the Children’s ECD programme in Zomba district, Malawi. The evaluation follows a mixed-methods approach (combining quantitative and qualitative methods) with two rounds of surveys and assessments timed 1 year apart, including child, caregiver, household, CBCC and market-level data collection.

### Study site

The intervention is targeted to disadvantaged communities within Zomba district in Malawi. The proposed study population includes CBCCs currently supported by Save the Children with an ECD package, including parenting and CBCC quality improvement. The geographical area for intervention was targeted by Save the Children on the basis of a set of education variables that impact pupil attendance and achievement in school. Within Zomba, two traditional authorities (TAs) and two sub-TAs were selected based on need (education and health) and the presence of other NGOs to implement the ECD programme. Save the Children’s ECD programme currently reaches 228 communities, 109 in TA Chikowi, 42 in TA Mbiza, 37 in sub-TA (STA) Ntholowa and 40 in STA Ngwelero. Sixty-eight of these (27 in TA Chikowi and 41 in TA Mbiza) had benefited from the agricultural and nutritional components already and were, therefore, excluded from the evaluation.

### Study population

The evaluation targets all children aged 0–6 years in the 60 selected communities and their caregivers. The primary reference group for this study is children aged 3–6 years old living in the service area of a Save the Children‐supported CBCC. Secondary reference groups include their siblings and caregivers.

### Random assignment and manipulation of treatments

The 60 communities were randomly assigned to one of two treatment arms:

1. Control group: communities with CBCCs supported by Save the Children’s ECD programme with no additional nutritional or agricultural support
2. Intervention group: communities with CBCCs supported by Save the Children’s ECD programme with additional nutritional and agricultural support

The integrated intervention package will be implemented in 30 of the 60 rural communities after the baseline survey and extended to the control communities after the end-line survey. There are several reasons why the control group in this case is not a control without intervention. The Government of Malawi is committed to scaling-up the ECD quality improvement across all CBCCs and an impact evaluation on the cost-effectiveness of different ECD quality improvement strategies is underway.^2^ The proposed evaluation complements the ongoing work by examining the relative impact and costs of alternative implementation models, focussing on how to enhance participation in the CBCCs and, at the same time, supporting the nutrition of children at a critical age in their development.

The 60 CBCCs were randomly selected in two stages from a pool of 235 CBCCs in 47 clusters currently assisted by Save the Children in Zomba. Due to the clustering of the CBCCs around primary schools, the list of 235 CBCCs was screened to flag clusters where more than one CBCC was being assisted. Twenty-six clusters were excluded from the first stage of randomisation to minimise possible contamination. An additional 10 CBCCs were dropped as they had ongoing activities. The 20 clusters were then randomly assigned to two groups of 10 clusters, where the randomisation was stratified geographically by traditional authority areas. In the second stage of randomisation, within each cluster, three CBCCs were then selected at random for the study. As six clusters had fewer than three CBCCs available for the study, in order to select a full sample of 30 CBCCs per treatment arm, additional CBCCs were randomly selected from three clusters (Gologota, St. Pius and Machereni).

### Sample sizes

**Fig. 2:**
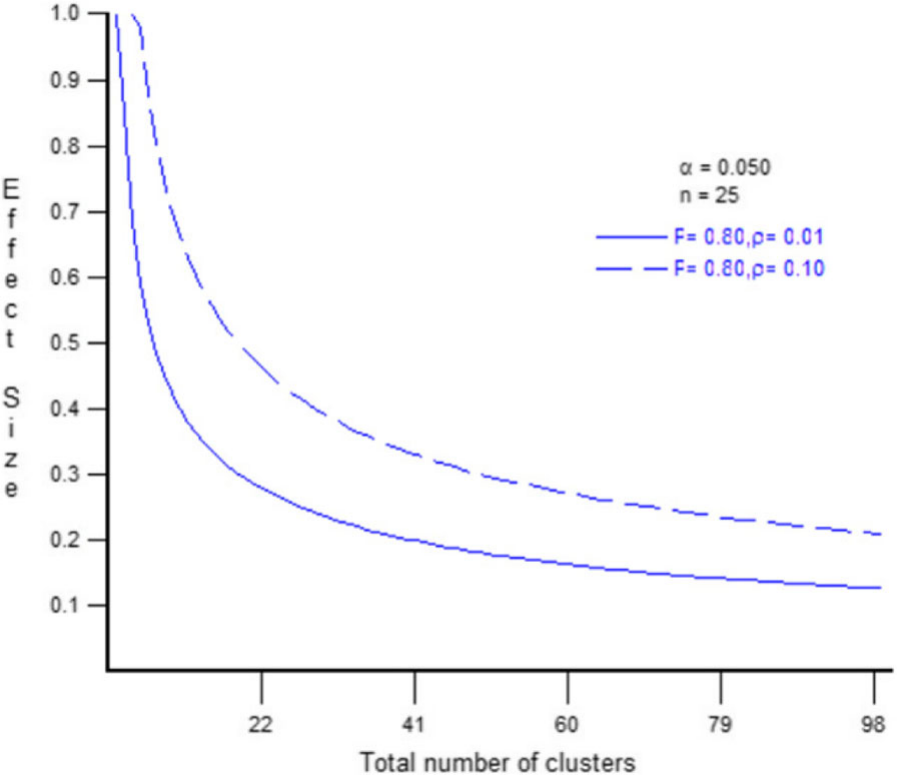
Diet diversity: minimum detectable effect size versus number of clusters, simulations with high and low intracluster correlation coefficients (ICCs)

Initial power calculations and resource availability had suggested the adoption of a sample with 30 clusters (communities) per treatment arm with 20 households in each cluster to identify reasonable treatment impacts of the intervention on the primary study outcomes. Data for power calculations was obtained from the 2010 Demographic and Health Survey (DHS) survey. We calculated means, standard deviations and intracluster correlation coefficients (ICCs) for rural children in Malawi. For Dietary Diversity Score (DDS), the mean and standard deviation for rural children aged 0–5 years were estimated to be 2.5 and 1.03, respectively. The ICC was 0.01. Plots of the standardised minimum detectable effect size (MDES) against the number of clusters assuming a sample of 25 children measured in each community (cluster size), consistent with 20 household interviews per community and considering that several children may end up not being tested, showed that only a marginal gain can be obtained by expanding the sample beyond 20 as power is mainly driven by the number of clusters [21] (see Fig. 2 for example simulations of MDES versus number of clusters with high and low ICCs).

**Fig. 3:**
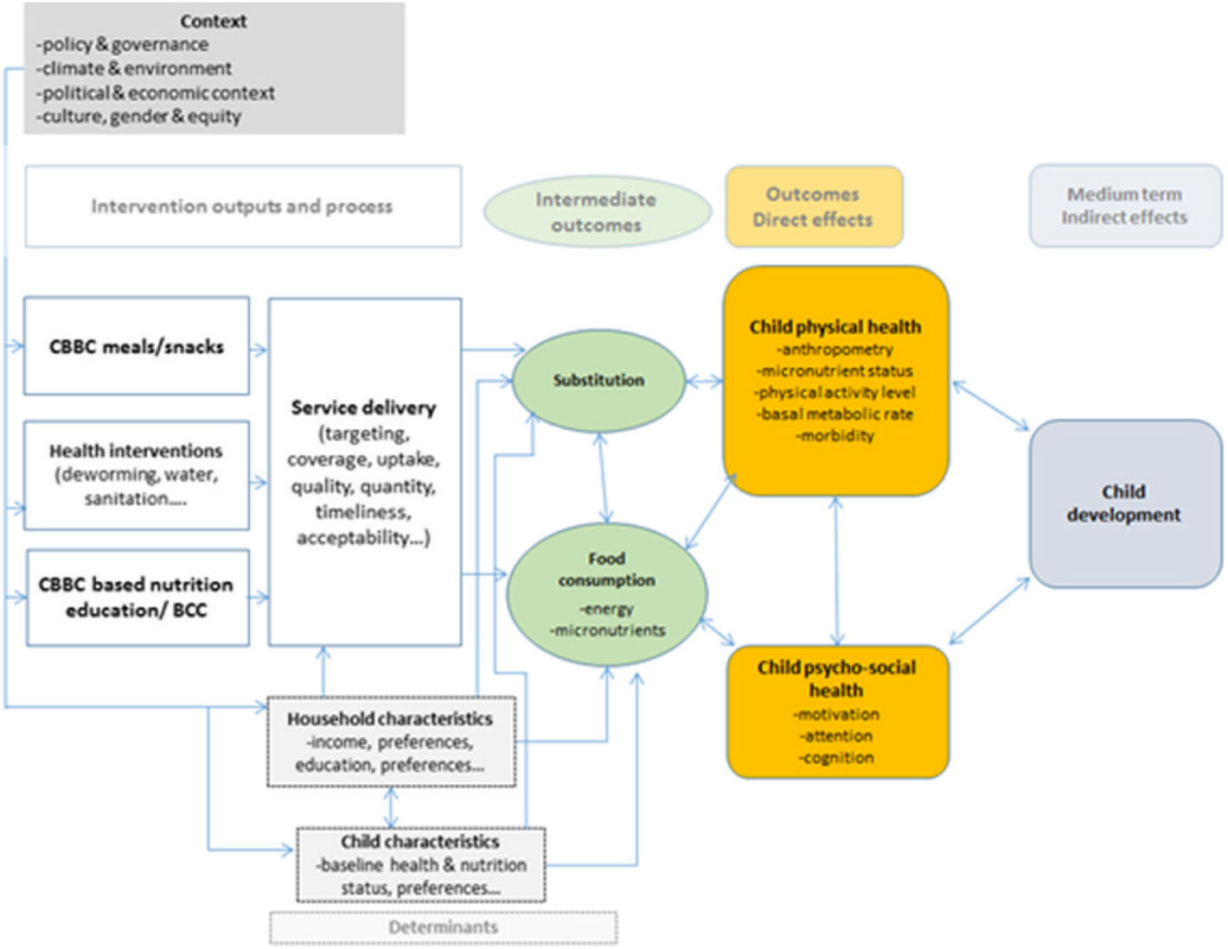
Impact pathways for the intervention on child nutrition and development (Fig. 3). (Source: adapted from [28])

After preliminary design visits to the targeted communities, the sampling strategy was modified to account for the implementation approach adopted by Save the Children, involving the clustering of CBCCs around surrounding primary schools. As a result, the cluster, or unit of randomisation was the primary school cluster that included a number of different CBCCs, rather than the CBCC itself. Adjusting for ICCs at the primary school cluster level, where 60 CBCCs were clustered into two groups of 15 primary school clusters, would provide 80% power to detect a 0.24-SD difference between treatment groups at the 5% level of significance (Table 3).

**Table 3:** Sample sizes

	Primary clusters	Communities/CBBCs	Households with children of target age	Children (0–5 y)	Children (3–5 y)
Control	15	30	600	936	648
Intervention	15	30	600	936	648
Total	30	60	1200	1872	1296

Note: Number of children estimated based on demographic data from the Demographic and Health Survey (DHS), 2010

*CBBCs* community-based childcare centres

The sampling of households was conducted through a census within a certain catchment area for each CBCC including information on the target age groups living within each household. Households with children aged 3–5 years were then randomly selected for participation in the survey.

### Data collection tools

The impact evaluation includes child-, caregiver-, household-, CBCC- and village-level data collection (Table 4). The household questionnaire collected data at the household level as well as for each relevant household member separately (main caregiver and all children in reference age groups).

**Table 4:** Survey questionnaire modules

Questionnaire	Module	Description
CBCC	Location and access	Identification, location
	Infrastructure	Physical infrastructure, including learning space, water and sanitation, cooking and storage facilities
	Staff	Staff roster, education and training
	Curriculum and services	Quality of CBCC activities and related services
	Caregivers health and nutrition knowledge	Knowledge related to optimal infant and young childcare and feeding practices
	Health and hygiene practices	Health and hygiene practices of CBCC staff
	Meal provision	Meal quality, portion sizes, meal planning, management and distribution
	Food procurement	List of food procured/sourced by the CBCC
	Garden land	Land used by CBCCs, including ownership and use, size of the plot, crops planted, input and labour for each plot
	Garden production	Crop production and use
	Garden sales	Crop sales, volumes and prices
	Food storage	Food storage infrastructure and practices
Household	Roster	Listing of demographic characteristics of household members
	Dwelling characteristics and assets	Basic features of the household’s primary dwelling place, including infrastructure, access to water and electricity
	Assets	Assets owned (by men and women separately)
	Land	Land owned and used by household’s women and men, including ownership and use, size of plot, crops planted, labour for each plot
	Agricultural production	Crop production and use
	Agricultural marketing	Crop sales, volumes, prices,
	Agricultural storage	Storage volumes and management
	Farm investments	On-farm investments and labour
	Farming practices	Pre- and post-harvest practices
	Livestock	Livestock holdings, revenue and costs, women ownership
	Employment and business enterprise	Non-farm sources of income (including employment), costs, male and female members
	Shocks	Unexpected events that may have influenced household’s wellbeing and responses taken by household
	Food security	Household vulnerability with respect to food frequency
	Food expenditures	Food expenditures and quantities consumed at household level
	Non-food expenditures	Expenditures on household items, clothing and personal expenditures over the last month
Caregiver	Caregiver’s health and hygiene	Health- and hygiene-related questions
	Caregiver’s health and nutrition knowledge	Knowledge related to optimal infant and young childcare and feeding practices
	Caregivers IYCF practices	Infant and child feeding practices
	Childcare practices	Childcare practices, including support for learning and stimulation
	Women’s time allocation	Women’s use of time, perceptions on women’s time use
Child	Child health	Child immunisation history and health-related questions
	Dietary assessment	Interactive 24-h recall on food intake for children aged 3–5 years
	Anthropometry	Physical measurements of all children and their parents
	Development	MDAT scores (fine and gross motor, language and social domains)

*CBCC* community-based childcare centre, *IYCF* Infant and Young Child Feeding, *MDAT* Malawi Development Assessment Tool

### Methods of analysis

The randomised design allows for the identification of causal impacts of interventions using comparisons of mean outcomes between the randomised treatment arms at end line. The analysis will follow the intention-to-treat approach as protocol and as treated, using econometric analysis for all the relevant outcomes of the intervention. Following [22], impact will be assessed using both a ‘difference-in-difference’ (DID) estimator and a single difference analysis of covariance (ANCOVA) model. Depending on the level of clustering of the outcome, we will employ multilevel regression models that account for the hierarchical nature of the data [23]. Multilevel models, also known as mixed-effects models, use both fixed effects (covariates) and random effects in at-school and household levels.

The DID estimate is calculated as the average change in the outcome of interest in the treatment arm minus the change in outcome in the control group. A difficulty of DID analysis involves serial correlation [15] resulting from unobserved factors affecting the outcomes that are themselves correlated over time. Serial correlation affects estimated standard errors and can lead to erroneous acceptance or rejection of null hypotheses but not the estimation of the effect size of the intervention. It may, therefore, lead to erroneously finding or not finding a statistically significant impact of the intervention. This problem can be addressed by calculating clustered standard errors [24]. Clustered standard errors will also be employed in all cases in which correlated outcomes are observed within the same unit of analysis. The analysis of covariance (ANCOVA) estimator has been shown to provide a more efficient estimate of programme impact when autocorrelation of outcomes is low [22].

### Heterogeneity of impact

The large dataset will allow for extensive subgroup analyses, including gender, age and geographic characteristics. The impacts of preschool feeding may be quite heterogeneous and context specific [24, 25]. School meals, for instance, have been associated with marked improvements in school participation of girls in rural areas where there are large gender disparities in access to education [26]. Furthermore, smallholder farmers targeted by the programme are mostly female.

### Cost-effectiveness

Cost data will be collected retrospectively following an ingredients approach using a semistructured questionnaire. The survey will be based on a standardised costing framework capturing capital (fixed) and recurrent costs incurred at the school level. The questionnaire will also cover both cash and in-kind contributions and will be used to estimate both financial and economic costs. Financial costs capture actual expenditures in terms of programme implementation on an annual basis. Economic costs included the opportunity costs of community members, teaching staff and other stakeholders involved in the intervention provision. Opportunity costs of preschool staff and community members will be calculated using local pay scales. Capital costs will be annuitised over the useful life of all relevant school-level assets using a discount rate of 3% as per World Bank recommendations. Annuitisation enables an equivalent annual cost to be estimated and reflects the value in-use of capital items, rather than reflecting when the item was purchased. Process and output data covering the adequacy of the service delivery will be collected from monitoring visits on a quarterly basis using standardised data collection forms. Output data will be combined with the costs to provide estimates of cost-efficiency metrics, including costs per beneficiary, kilocalories, iron, and vitamin A delivered. Sensitivity analysis will be undertaken to account for uncertainties in the economic evaluation. The figures obtained in this way will then be compared to figures calculated for other interventions.

### Data collection

The enumerators will be recruited from Chancellor College, University of Malawi and trained for the baseline survey. Each team, led by a supervisor and assisted by community leaders, will conduct household listings and sampling in each enumeration area. The data collection will be undertaken using electronic tablets. Data collection will be reviewed daily by a team supervisor and inconsistencies clarified. Dietary assessment will be undertaken by trained and supervised enumerators using the interactive 24-h recall method. Prior to the recall interview, caregivers will be briefed on the purpose and methods of interview. The interview will be conducted using visual aids to assist in estimating portion sizes of the foods consumed. The 24-h dietary assessment will be repeated on nonconsecutive days for a subset of households (approximately 20%) to obtain estimates of usual intake [27]. Anthropometry collection will include measurements of children’s height and weight. Height or length will be measured to the nearest 0.1 cm using portable fixed base stadiometers and weight will be measured to the nearest 0.1 kg using electronic scales. The height and weight measures will be assembled and placed on a level surface. In the absence of a level ground in the household, a suitable place will be identified for the measurement in the community. Training on the MDAT will be provided to all supervisors and enumerators by trained staff from the College of Medicine, Malawi. During the MDAT training all enumerators will be reviewed for consistency and reliability.

## Discussion

Early childhood development programmes are considered as the most cost-effective form of human capital investment compared to any subsequent schooling interventions [4]. Emerging evidence highlights the potential additive and synergistic effects combining ECD, and nutrition [6, 7], and agriculture and nutrition [3]. Combining these three sectors may lead to substantial gains in cost, efficiency and effectiveness; however, there is a need to rigorously assess these potential synergies.

This paper describes the design for a CRT of a preschool-feeding-based intervention linked to smallholder agricultural and community engagement. As far as we are aware, it is the first to explicitly examine (from a food systems perspective) the simultaneous impact of a preschool meals programme on dietary choices, alongside outcomes in the nutritional, child development and agricultural domains. As the intervention is complex, the scope of this theory-based evaluation includes measurement of a range of outcome and process metrics across multiple disciplines. The data collection requires multisectoral expertise in the survey teams, including measurements of dietary intake, anthropometry and child development, alongside expenditure and other socioeconomic-related dimensions.

### Trial status

**Fig. 4:**
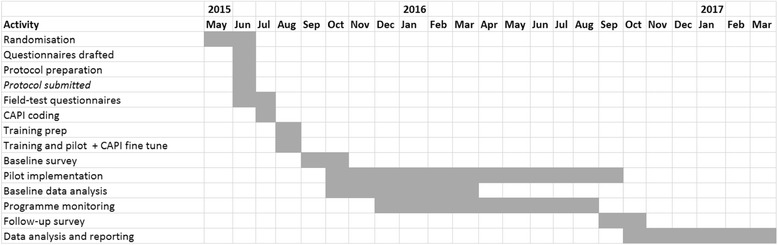
Study timeline

The first participant was enrolled on 7 September 2015. Data collection for the last of 120 participants will be completed by December 2016 (see the SPIRIT Checklist figure (Additional files 1 and 2)) (Fig. 4).

### Additional files

Additional file 1: The schedule of enrolment, interventions and assessments for the NEEP-IE study. (DOCX 14 kb)

Additional file 2: SPIRIT 2013 Checklist. (DOC 120 kb)

## Abbreviations

ANCOVA: Analysis of covariance
CBCCs: Community-based childcare centres
CBO: Community-based organisation
CRT: Cluster randomised trial
DDS: Dietary Diversity Score
DID: Difference-in-difference
ECD: Early Childhood Health and Development
FVS: Food Variety Score
HANCI: Hunger and Nutrition Commitment Index
HAZ: Height for age z-score
HH: Household
IRI: Interactive radio instructions
IYCF: Infant and Young Child Feeding
MDAT: Malawi Development Assessment Tool
MDES: Minimum detectable effect size
NEEP-IE: Nutrition Embedded Evaluation Programme Impact Evaluation
OFSP: Orange-fleshed sweet potato
SUN: Scaling Up Nutrition
TA: Traditional Authority
WALA: Wellness and Agriculture for Life Advancement

## Footnotes

1 Save the Children, unpublished.

2 The Protecting Early Childhood Development (PECD) trial.

## References

[1] Black RE, Victora C, Walker SP, Bhutta ZA, Christian P, De Onis M (2013). Maternal and child undernutrition and overweight in low- and middle-income countries. Lancet.

[2] Masset E, Haddad L, Cornelius A, Isaza-Castro J (2011). A systematic review of agricultural interventions that aim to improve nutritional status of children.

[3] Ruel MT, Alderman H, and the Maternal and Child Nutrition Study Group (2013). Nutrition-sensitive interventions and programs: how can they help to accelerate progress in improving maternal and child nutrition?. Lancet.

[4] Heckman JJ (2006). Skill formation and the economics of investing in disadvantaged children. Science.

[5] Alderman H (2010). The economic cost of a poor start to life. J Dev Orig Health Dis.

[6] Engle PL, Black MM, Behrman JR, Cabral de Mello M, Gertler PJ, Kapiriri L (2007). Strategies to avoid the loss of developmental potential in more than 200 million children in the developing world. Lancet.

[7] Grantham-McGregor SM, Fernald LC, Kagawa RM, Walker S (2014). Effects of integrated child development and nutrition interventions on child development and nutritional status. Ann NY Acad Sci.

[8] von Grebmer K, Bernstein J, de Waal A, Prasai N, Yin S, Yohannes Y. 2015 Global hunger index: Armed conflict and the challenge of hunger. Bonn, Washington, and Dublin: Welthungerhilfe, International Food Policy Research Institute (IFPRI) and Concern Worldwide; 2015.

[9] National Statistical Office/Malawi and ICF. Malawi Demographic and Health Survey 2015-16. Zomba: National Statistical Office and ICF; 2017.

[10] Neuman MJ, McConnell C, Kholowa F (2014). From early childhood development policy to sustainability: the fragility of community-based childcare services in Malawi. Int J Early Child.

[11] Scaling-Up Nutrition (SUN) Country Profile Malawi. http://scalingupnutrition.org/sun-countries/malawi. Accessed 06 June 2017.

[12] Gillespie S, Harris L, Kadiyala S (2012). The agriculture-nutrition disconnect in India: what do we know? Discussion Paper 01187.

[13] Webb P (2013). Impact pathways from agricultural research to improved nutrition and health: literature analysis and research priorities.

[14] Turner R, Hawkes C, Jeff W, Ferguson E, Haseen F, Homans H (2013). Agriculture for improved nutrition: the current research landscape. Food Nutr Bull.

[15] Arimond M, Wiesmann D, Becquey E, Carriquiry A, Daniels MC, Deitchler M (2010). Simple food group diversity indicators predict micronutrient adequacy of women’s diets in 5 diverse, resource-poor settings. J Nutr.

[16] Working Group on Infant and Young Child Feeding Indicators. Developing and Validating Simple Indicators of Dietary Quality of Infants and Young Children in Developing Countries: Additional analysis of 10 data sets. Report submitted to the Food and Nutrition Technical Assistance Project )17/FHI 360, September 2007.

[17] Bhutta ZA, Das JK, Arjumand R, Gaffey MF, Walker N, Horton S (2013). Evidence-based interventions for improvement of maternal and child nutrition: what can be done and at what cost?. Lancet.

[18] Polliltt E, Gersowitz M, Gargiulo M (1978). Educational benefits of the United States school feeding program: a critical review of the literature. Am J Public Health.

[19] Grantham-McGregor SG, Ani G (2001). Iron-deficiency anemia: reexamining the nature and magnitude of the public health problem. J Nutr.

[20] Masset E, Gelli A (2013). Community participation and the links between agriculture, nutrition and education: design of a randomised field experiment of ‘home-grown’ school feeding in Mali. Trials.

[21] Hayes R, Moulton LH (2009). Cluster randomised trials.

[22] Bruhn M, McKenzie D (2009). In pursuit of balance: randomization in practice in development field experiments. World Bank Policy Research Working Paper 4572.

[23] Goldstein H (2003). Multilevel statistical models.

[24] Angrist JD, Pischke JS (2009). Mostly harmless econometrics.

[25] Kristjansson EA, Robinson V, Petticrew M, MacDonald B, Krasevec J, Janzen L (2007). School feeding for improving the physical and psychosocial health of disadvantaged elementary school children. Cochrane Database Syst Rev.

[26] Gelli A (2015). School feeding and girls’ enrollment: the effects of alternative implementation modalities in low-income settings in sub-Saharan Africa. Front Public Health.

[27] Willett W (2013). Nutritional epidemiology.

[28] Gelli A, Espejo F, Shen J, Kristjansson E (2014). Putting it all together: aggregating impacts of school-feeding programmes on education, health and nutrition: two proposed methodologies’. WIDER Working Paper 2014/036.

