# Supplementary material for: Improving child nutrition and development through community-based childcare centres in Malawi – The NEEP-IE study: study protocol for a randomised controlled trial
Source: Trials. 2017 Jun 19;18:284. doi: 10.1186/s13063-017-2003-7 (PMC5477384; doi:10.1186/s13063-017-2003-7)
Supplement: Supplementary file 1 — The schedule of enrolment, interventions and assessments for the NEEP-IE study. (DOCX 14 kb) [file 13063_2017_2003_MOESM1_ESM.docx]

The schedule of enrolment, interventions, and assessments for the NEEP-IE study.

|  | **STUDY PERIOD** | | | | | | | |
| --- | --- | --- | --- | --- | --- | --- | --- | --- |
|  | **Enrolment** | **Allocation** | **Post-allocation** | | | | | **Close-out** |
| **TIMEPOINT**** | ***-t_1_*** | **0** | ***t_1_*** |  |  |  |  | ***t_2_*** |
| **ENROLMENT:** |  |  |  |  |  |  |  |  |
| **Eligibility screen** | X |  | X |  |  |  |  |  |
| **Informed consent** | X |  | X |  |  |  |  |  |
| **Allocation** |  | X | X |  |  |  |  |  |
| **INTERVENTIONS:** |  |  |  |  |  |  |  |  |
| ***Agriculture-nutrition intervention*** |  |  | X |  |  |  |  |  |
| ***Parenting and childcare centre training*** |  |  | X |  |  |  |  |  |
| **ASSESSMENTS:** |  |  |  |  |  |  |  |  |
| ***Baseline survey (all outcomes and covariates)*** | X |  |  |  |  |  |  |  |
| ***Endline survey (all outcomes and covariates)*** |  |  |  |  |  |  |  | X |
